# Potential Therapeutic Roles of Exosomes in Multiple Myeloma: A Systematic Review

**DOI:** 10.7150/jca.31752

**Published:** 2019-10-15

**Authors:** Mengzhen Li, Bing Xia, Yi Wang, M. James You, Yizhuo Zhang

**Affiliations:** 1 Department of Pediatric oncology, Sun Yat-sen University Cancer Center, State Key Laboratory of Oncology in South China, Collaborative Innovation Center for Cancer Medicine, Guangzhou, Guangdong 510060, China; 2 Department of Hematology, Tianjin Medical University Cancer Institute and Hospital, National Clinical Research Center for Cancer; Key Laboratory of Cancer Prevention and Therapy, Tianjin; Tianjin's Clinical Research Center for Cancer; 300060, China; 3 Department of Hematopathology, The University of Texas MD Anderson Cancer Center, Houston, Texas, USA

**Keywords:** exosomes, multiple myeloma, potential therapeutic target, miRNA, protein, drug delivery

## Abstract

Multiple myeloma (MM) is the second most prevalent hematological malignancy. In spite of the remarkable progress in understanding the biology and therapy of MM, curing this disease remains difficult, which calls for more effective treatment strategies. As vital communicators between different cells, exosomes have been verified to be crucial to cancer diagnosis, treatment, and prognosis. Exosomes in MM patients show a different expression profile compared with those in healthy individuals. In this review, we summarize potential therapy roles exosomes may play in MM. The specific expression of certain components in exosomes may provide therapeutic targets. Moreover, tumor-derived exosomes and their modified products can be developed into vaccines for anti-tumor immunity. In addition, the natural nano structure of exosomes makes them excellent carriers for drug delivery. Thus, a more rigorous investigation into exosomes will pave the way for novel tumor therapies in MM patients.

## Introduction

Multiple myeloma, which features with constant clonal expansion of plasma cells in bone marrow (BM), is the second most prevalent hematological malignancy and is currently incurable. Although current treatment strategies, including chemotherapy, immunotherapy, and autologous stem cell transplantation can improve the survival of MM patients, many patients still experience recurrent-refractory disease. Hence, there is an urgent need to explore new therapeutic approaches for MM.

The structure and function of exosomes have been studied since their first discovery in the twentieth century. Exosomes are membranous vesicles that are constitutively released by almost all kinds of cells. They were first coined by Trams et al to describe the extracellular release of vesicles with 5' -nucleotide activity by normal cells and tumor cells 1. Then in the late 1980s, this term was used to describe vesicles of endosomal origins released after the fusion of multivesicular bodies (MVBs) with cytoplasmic membranes during reticular cell differentiation 2. Nowadays exosomes are proven to play key roles in regulating both physiological and pathological activities among cells. In addition to mediating local cell-to-cell communication by transferring proteins, lipids, and nucleic acids to recipient cells, exosomes can also induce the activation, proliferation, differentiation, and death of target cells. Some components of exosomes such as miRNAs and proteins that are specifically expressed in MM may be useful as therapeutic targets. Using their inhibitors or analogs to stabilize specific exosome expression levels in MM patients may decrease the carcinogenic effects or increase the tumor-suppressive effects of exosomes. Tumor-derived exosomes and their modified products can be developed into vaccines for anti-tumor immunity. Furthermore, natural nano structures of exosomes make them proper carriers to deliver target drugs.

## Overview of exosomes

Exosomes are a kind of extracellular vesicles (EVs). On the basis of differences in biosynthesis and size, EVs are divided into three subgroups: microparticles derived from the shedding membrane, exosomes arising from MVBs, and EVs resulting from apoptosis. Microparticles (also known as microvesicles) are medium-sized vesicles, ranging from 200 to 1,000 nm; apoptotic bodies are larger (800-5,000 nm), whereas exosomes are smaller (30-150 nm) 3. Exosomes are generated through endocytosis pathways in which the cell membrane sinks into the inner body to form early endosomes. Then early endosomes developed into late endosomes, which are transformed into polycystic bodies with a dynamic subcellular structure (MVB) 4. MVBs are important protein transport and sorting centers of eukaryotic cells and are closely related to signal conduction, cytoplasmic division, gene silencing, autophagy, and virus budding. When MVBs fuse with lysosomes, the inner luminal vesicles undergo degradation and then fuse with the plasma membrane. Next, the inner luminal vesicles within sunk again, budding into granular vesicles known as exosomes 5, and then are released into the extracellular environment and body fluids (plasma, urine, saliva, ascites, amniotic fluid, lactic and cerebrospinal fluid) 6.

Exosomes can be secreted by almost all kinds of cells and act as signal presenters in the microenvironment. The exosome surface is rich in cholesterol, sphingomyelin, ceramide lipids and contains protein, mRNA, miRNA, and other biological information, which can be delivered to various recipient cells. There are several ways by which exosomes are taken up by recipient cells: (a) receptor-or lipiodraft-mediated endocytosis, (b) phagocytosis, (c) macropinocytosis, or (d) fusion with the plasma membrane of a target cell 7.

## Influence of exosomes on MM

**Figure 1:**
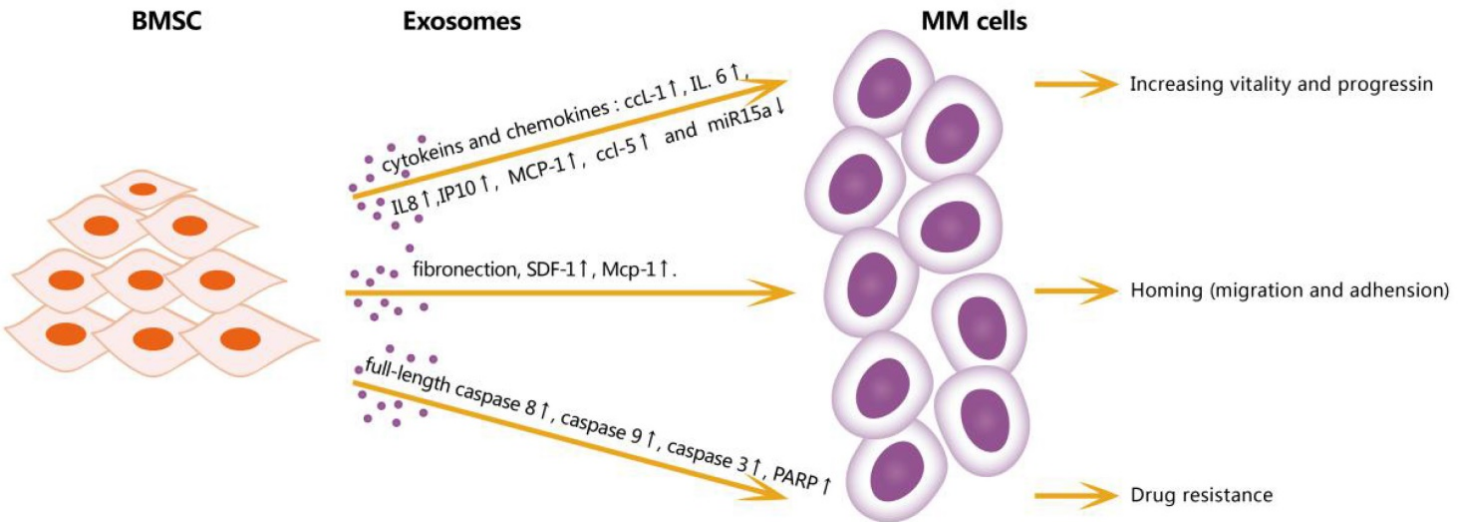
** Effects of exosomes from BMSC in microenviroment on MM cells.** BMSC-derived exosomes can increase vitality and progression of MM cells through cytokines and chemokines: IL-6, IPIO, MCP-1, CCL1 and downregulate miR15a; increase homing by upregulating fibronection, SDF1 and MCP-1; promote and resistance by increasing full-length caspase-8, caspase9, caspase3 and PARP. CCL1: chemokine ligand 1, IL6: interleukin 6, IL8: interleukin 8, IP10: interferon - inducible protein -10, MCP-1: monocyte chemotactic protein-1, CCL5: chemokine ligand 5, SDF1: stromal cell-derived factor 1, PRAP: poly ADP-ribose polymerase.

The BM microenvironment is closely associated with MM generation, progression, immunosuppression, and drug resistance. BM stromal cells (BMSCs) may play important roles in the BM microenvironment, but these roles have not been fully elucidated [Figure 1]. One study demonstrated a tumor-supportive role of BMSC-derived exosomes: compared with BMSC-derived exosomes in normal samples, BMSC-derived exosomes in MM expressed a lower level of miRNA-15a, which has been identified as a tumor-suppressive factor. In addition, BMSC-derived exosomes in MM expressed a higher level of fibronectin, indicating that they affect MM cell adhesion differentially 8. Another study by Wang et al indicated that BMSC-derived exosomes can promote the proliferation, migration, and survival of MM cells, induce drug resistance to bortezomib, and influence several signaling pathways including c-Jun N-terminal kinase, TP38, TP53, and AKT, which are relevant to the survival of MM cells 9.

**Figure 2:**
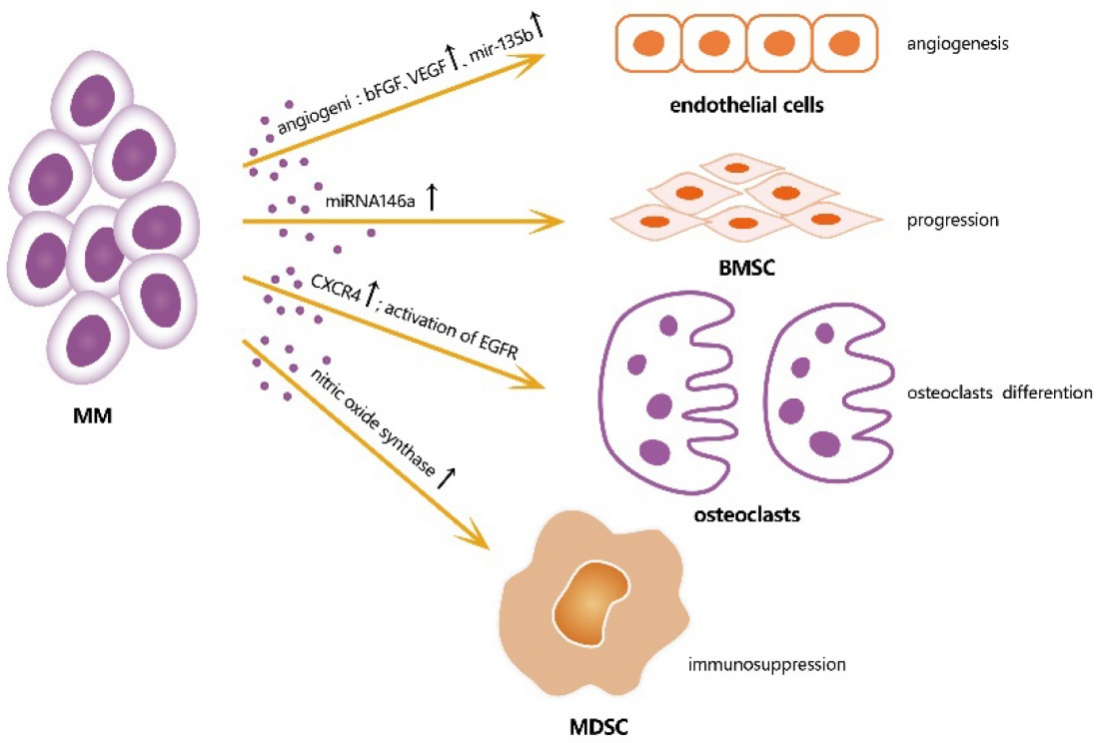
** Effects of exosomes from MM cells on different cells in microenvironment, thus induce a favorable microenvironment for MM growth.** bFGF: b fibroblast growth factor; VEGF: vascular endothelial growth factor; CXCR4: cysteine X cysteine receptor 4; EGFR: epidermal growth factor receptor ligands.

Accordingly, MM-derived exosomes influence the activities of various cells in the BM microenvironment by creating a pro-tumor environment for MM [Figure 2]. A study showed that miRNA-146a in MM-derived exosomes could be transmitted into BMSCs, resulting in the up-regulation of some specific cytokines and chemokines, such as CXCL1, IL-6, IL-8, IP10, MCP-1, and CCL-5, which leaded to the increased vitality and progression of MM cells. This study showed a positive feedback loop in which MM cells up-regulated the expression of miRNA-146a in BMSCs, resulting in more cytokine secretion, which in turn favored MM cell growth and migration 10. A study of angiogenesis and immunosuppression, both *in vivo* and *in vitro*, demonstrated the presence of multiple angiogenic factors, such as angiogenin, basic fibroblast growth factor, vascular endothelial growth factor, and miRNA-135b, were closely associated with angiogenesis in MM 11. Umezu et al also demonstrated that miRNA-135b from MM-derived exosomes accelerated HIF-1 transcriptional activity by inhibiting FIH-1; this HIF-FIH signaling pathway exerted an angiogenesis influence 12. In addition, MM-derived exosomes can serve as a promoter for MM immunosuppression. Exosomes up-regulate nitric oxide synthase in myeloid-derived suppressor cells (MDSCs) and enhance their immunosuppression ability 13. MM-derived exosomes can also modulate the survival and differentiation of osteoclasts by increasing CXCR4 expression and triggering the AKT pathway 14. Stefania Raimondo et al also demonstrated that MM-derived exosomes were enriched of amphiregulin (AREG), and this exosomes-derived AREG can led to the activation of epidermal growth factor receptor ligands (EGFR) in preosteoclast, thus help to induce differentiation of osteoclasts 15. Overall, exosomes serve as information communicators between cells and play a vital role in MM progression.

## miRNA related potential treatment targets for MM in exosomes

Exosomes can be potential diagnostic biomarkers and facilitate the diagnosis and classification of diseases and contribute to the prediction of prognosis in cancer patients as well 16-19. As an important component of exosomes, miRNAs vary in types and amounts among different exosomes and cells, and the abnormal expression of certain miRNAs is often associated with the clinical diagnosis. However, there are limited studies about miRNAs in MM therapy.

One important reason that studies of miRNAs in MM are limited is the lack of reliable and relatively simple technologies to separate and extracting specific miRNAs from samples. Another reason is that the therapeutic application of miRNAs is still under clinical investigations. The highly effective delivery of suitable miRNAs to MM cells and their uptake by the latter with no off-target effects remain challenging, hindering the development of an effective miRNA-based therapy. Nevertheless, the abnormal expression of certain miRNAs in MM-derived exosomes seems to be a treatment target in MM [Table 1].

As mentioned before, the high expression of miRNA-146a, promotes tumor progression through its involvement in the endogenous Notch pathway. The Notch pathway interacts with the NF-κB, JAK-STAT, and MAPK pathways, and they work together to regulate multiple gene transcriptions in MM progression. Therefore, DAPT, an inhibitor of endogenous Notch pathway, can inhibit the release of cytokines induced by miRNA-146a in MM 10. miRNA-135b is expressed highly and exists stably in the hypoxic MM microenvironment, promoting endothelial vessel formation through the HIF-FIH signaling pathway. miRNA-135b may only be related to local area information transmission instead of cyclic plasma cells. Therefore, miRNA-135b may not be a suitable biomarker for MM diagnosis. However, its role in local tumor angiogenesis makes it a biomarker of angiogenesis in MM patients with locally relapsed disease, which inspires the exploration of specific blocking agents of miRNA-135b that may inhibit local angiogenesis 12.

miRNAs regulate gene expressions. Targeting the miRNA that act as tumor suppressors seems to be a promising miRNA-based treatment strategy. A recent study demonstrated that miRNA-34a over-expression in MM cells inhibited both cell proliferation and colony formation while increasing apoptosis in cancer stem cells *in vitro* 20. Transient expression of miRNA-34a synthetic mimics or lentivirus-based miRNA-34a transduced into mice induced growth suppression and apoptosis in MM cells. Synthetic miRNA-34a downregulated the transcription and translation of BCL2, CDK6, and Notch 1. In addition, the anti-MM activity of miRNA-34a was found to result in no evidence of toxicity in mice, suggesting a favorable therapeutic index 21.

In a study by Leone, E. et al, significant inhibition of primary MM cells or of IL-6-dependent and -independent MM cell proliferation was triggered by miRNA-21 inhibitors. Meanwhile, transfection of miRNA-21 mimics increased the proliferation of MM cells, demonstrating the tumor-promoting potential of miRNA-21 in MM. Therefore, transient enforced expression or lentivirus-based constitutive expression of miRNA-21 inhibitors appears to be a promising treatment strategy for MM 22. Morelli et al showed that anti-MM activity was induced by selectively targeting interferon regulatory factor 4 with synthetic miRNA-125b-5p mimics *in vitro* and *in vivo*. The fact that interferon regulatory factor 4 was inhibited, and its downstream effectors—c-MYC, CASPASE-10, and c-FLIP—were down-regulated as well, provided proof-of-concept that synthetic miRNA-125b-5p mimics are promising anti-MM agents 23. Another study revealed a novel role of miRNA-137/197 in mediating MM cell apoptosis by targeting MCL-1. Lentivirus-based or formulated synthetic miRNA-137/197 exerted a therapeutic effect in preclinical models, which supported the suppression role of miRNA-137/197 in MM 24.

Upregulated miRNAs may be MM therapeutic targets by blocking their promoting effect on MM growth with appropriate inhibitors. Down-regulated miRNAs can be targeted using lentivirus-based or formulated synthetic mimics or other mimics. Nevertheless, one of the challenges in miRNA therapy is that mimics must be confirmed to have acceptable side effects or toxicities, which necessitates more clinical trials. In addition, more advanced technologies are needed for the mass production of mimics.

## Protein related potential treatment targets for MM in exosomes

Allogenic exosomes have the potential to overcome one of the main challenges of cell-based therapies—immune response. They can provide a relatively stable environment for therapeutic agents and show the potential to be modified to enhance cell-specific homing, as well as to fuse with the plasma membranes of cells, making it possible for agents to enter the target cells directly 25. The potential therapeutic role of proteins derived from exosomes in MM has been attractive. However, the unstable variety and content of proteins due to individual and organ tissue specificity have prevented the further study of exosomes-derived proteins. In addition, the protein may not be a suitable surrogate for EV quantity since each EV contains various amounts of proteins. Nevertheless, exosome-derived proteins of MM show significant value as biomarkers for diagnosis and prognosis. High expression of specific proteins in exosomes from different body fluids can be used to facilitate the diagnosis and predict the prognosis of a variety of diseases, such as lung cancer, pancreatic cancer, liver cancer, cholangiocarcinoma, gastric cancer, nervous system diseases, and genitourinary system diseases 26-27.

Currently, a protein named heparanase, which is delivered through the secretion of exosomes, is attracting the attention of many researchers. Released by tumor or host cells such as macrophages, heparanase can diffuse within the microenvironment and play a part in the functions of neighboring tumor cells by interacting with them and promoting the secretion of exosomes. It impacts exosome protein cargo by inducing it to express cytokines that are related to the development of tumors and the formation of blood vessels, such as syndecan-1, vascular endothelial growth factor, and hepatocyte growth factor at higher levels compared with heparenase-low expressing cells 28-29. Studies have shown that compared with healthy donors, the expression of heparanase in MM patients is higher, indicating that heparanase is a therapeutic target in these patients [Table 1]. A study by Ritchie et al demonstrated that a heparanase inhibitor called SST0001 disrupted the MM tumor microenvironment and inhibited tumor growth 30. Another study demonstrated that H1023, a monoclonal antibody, inhibited heparanase enzymatic activity 31. The inhibitors of heparanase that are currently being evaluated in clinical trials are all modified heparins or heparin mimics; more types of highly specific inhibitors, such as monoclonal antibodies and small molecule chemical inhibitors, have yet to be exploited. Further studies are also needed to explore the exact composition and characteristics of proteins in MM-derived exosomes and to identify suitable regulators that react with proteins to make it possible for exosome-derived proteins to be utilized in clinical therapy.

## Other exosome-associated treatment methods for MM

Apart from miRNAs and proteins, there are other exosome-associated treatment methods for MM [Table 1]. For instance, C6 ceramide, an exogenous ceramide supplement, was found to dose-dependently stimulate exosome secretion and elevate the exosome levels of some tumor-suppressive miRNAs, such as miRNA-202, miRNA-16, miRNA-29b, and miRNA-15a, thus increasing the apoptosis and inhibiting the proliferation of recipient MM cells, indicating that it is a target for MM treatment 32. Exosomes also show anti-tumor immune effects. For example, a previous study confirmed that exosomes secreted by dendritic cells induced the inhibition of tumor growth in mice. In this study, the authors reported that the treatment efficacy of dendritic cells from the peripheral blood of stage III/IV non-small-cell lung cancer (NSCLC) and melanoma patients who did not undergo surgery was evaluated successfully in a phase I clinical trial, and no CD8^+^ T cells were detected in response 33.

According to a study by Xie et al, vaccines developed from exosomes were effective for MM. In their studies, exosomes from MM cells were used to stimulate anti-tumor immune responses and produce prophylactic immunity in MM cell lines 34-35. A membrane protein named multiple myeloma special antigen-1, was specifically expressed in and correlated with MM. By taking advantage of its epitope, a vaccine called SLSLLTIYV, induced the obvious cytotoxic T lymphocyte response *in vitro*. MM special antigen-1-derived epitopes could combine with Dickkopf-1 to construct a multi-epitope peptide vaccine that substantially improved the survival rate compared with a single-epitope vaccine in a severely immune-deficient MM mouse model. This multi-epitope vaccine also greatly relieved the tumor burden and alleviated bone destruction, which will benefit MM patients in individualized therapy.

## Exosomes as proper carriers to deliver target drugs

In addition to providing potential therapeutic targets, the natural nano structures of exosomes enable them excellent to be drug carriers and beneficial tools for tumor-targeted drug therapy. The surface of exosomes is a phospholipid bilayer mounted with a complex and special protein, which provides effective protection for loaded drugs within exosomes and helps these drugs to escape the capture by the reticuloendothelial system, extending their circulation time to combine with targets *in vivo*. More importantly, exosomes membranes can easily fuse with their counterparts in receptor cells, allowing the loaded drugs to be released into the cells directly. This natural structure of exosomes makes them perfect carriers for transporting drugs, such as gene drugs, anti-cancer drugs, and anti-inflammatory drugs 36-41. According to a reported study, when the researchers developed a formulation of paclitaxel (PTX) -loaded exosomes with incorporated aminoethylanisamide-polyethylene glycol (AA-PEG) vector moiety in order to target the sigma receptor, which is overexpressed by lung cancer cells, the AA-PEG-vectorized exosomes loaded with PTX (AA-PEG-exoPTX) were found to possess a high loading capacity, strong ability to accumulate in lung cancer cells upon systemic administration, and also improved therapeutic outcomes. The combination of targeting ability with the biocompatibility of exosome-based drug formulations offers a novel delivery choice for anticancer therapy 42. At present, there are several methods for loading “cargo” into exosomes: (a) transfection of exosome‑secreting cells 43, (b) chemical‑based exosome incorporation with exogenous cargoes 44, (c) electroporation 45 and alternative exosome‑loading techniques 46.

Exosome mimetics have similar sizes, surface morphologic features, and targeting abilities as exosomes. Jang et al discovered that chemotherapeutic drug-loaded nanovesicles, as exosome mimetics, inhibited tumor growth *in vitro* and *in vivo*, with no adverse effects 47; Wu et al first reported the preparation for exosome-mimetic nanovesicles (NVs) from primary hepatocytes with almost 100 times the production yield compared with exosomes. Meanwhile, NVs were demonstrated to have both components and biofunctions similar to exosomes from primary hepatocytes 48. These findings indicate that exosome mimetics, serving as drug carriers to replace exosomes, can effectively overcome two common issues with exosomes: the difficulties of mass production and the time-consuming nature of the purification process. Therefore, exosomes have many advantages over other existing delivery systems. In MM therapy, it may be possible to use exosomes or their mimics as carriers to load miRNA or protein inhibitors or mimics, since endogenous miRNAs can be encapsulated into exosomes to escape endonuclease degradation.

## Conclusions and future perspective

Taken together, several abnormally expressed components in MM-derived exosomes, including miRNAs, proteins, and sphingolipids are potential targets for MM treatment. Utilizing the regulators of these components may have a tumor-suppressive effect. In addition, exosomes can be developed into vaccines for prophylactic immunity. The natural nano structure of exosomes makes them excellent carriers for drug delivery. However, when it comes to the future use of exosomes, there are still issues that need to be resolved. For components such as miRNAs, proteins, and sphingolipids, mass production of their mimics or regulators remains a major obstacle. Studies of exosome vaccines are still limited. Thus, more studies are needed to ensure that these treatments are effective and safe. Satisfactory efficacy and safety profiles have been demonstrated for exosomes and their mimics in other types of diseases, including NSCLC, type 1 diabetes mellitus, and colon cancer, but clinical trials in MM are still absent. Therefore, MM clinical trials are indispensable.

## Figures and Tables

**Table 1:** Exosome-related therapy targets for MM patients

Target	Property in exosomes	Source	Expression pattern	Function	Ref.
miR-15a	miRNA	BM-MSCs	Down-regulated	Inhibit MM cell proliferation	8
miR-146a	miRNA	BM-MSCs	Up-regulated	Stimulate the production of more cytokines that promote the growth of MM cells, including CXCL1, IL-6, IL-8, IP10, MCP-1, and CCL-5	10
miR-135b	miRNA	MM cells	Up-regulated	Suppress factor-inhibiting hypoxia inducible factor 1 (FIH-1) in endothelial cells directly	12
miR-34a	miRNA	MM cancer stem cells (CSCs)	Down-regulated	Inhibit cell proliferation, colony formation, and increase CSC apoptosis *in vitro*	20
miR-34a	miRNA	MM cells	Down-regulated	Trigger growth inhibition and apoptosis in MM cells	21
miR-21	miRNA	MM cells	Up-regulated	MiR-21 inhibitors trigger significant growth inhibition of primary MM cells or IL-6-dependent/-independent MM cells	22
miR-125b-5p	miRNA	MM cells	Down-regulated	Impair MM cell growth and survival *in vitro*	23
miR-137/197	miRNA	MM cells	Down-regulated	Reduce MCL-1 protein expression, alter apoptosis-related gene expression, and induce apoptosis and inhibit viability, colony formation, and migration in MM cells	24
Heparanase	Protein	MM cells	Up-regulated	Regulate tumor metastasis, angiogenesis, and chemoresistance	29
STT0001	Chemically modified heparin	--------	--------	Inhibit myeloma growth *in vivo* effectively and cause changes within tumors that are consistent with the compound's ability to inhibit heparanase	30
Ceramide	Sphingolipid	--------	--------	Dose-dependently inhibit proliferation and promote apoptosis in human MM OPM2 cells and increase exosomal levels of tumor-suppressive miRNAs (miR-202, miR-16, miR-29b, and miR-15a)	32

## Abbreviations

MM: multiple myeloma
MVBs: multi-vesicle bodies
EVs: extracellular vesicles
BM: bone marrow
BMSCs: bone marrow stromal cells
MDSCs: myeloid-derived suppressor cells
AREG: amphiregulin
EGFR: epidermal growth factor receptor
NSCLC: non-small-cell lung cancer
PTX: paclitaxel
AA-PEG: aminoethylanisamide-polyethylene glycol
AA-PEG-exoPTX: AA-PEG-vectorized exosomes loaded with PTX
NVs: nanovesicles
